# Vitamin C Deficiency Exacerbates Dysfunction of Atherosclerotic Coronary Arteries in Guinea Pigs Fed a High-Fat Diet

**DOI:** 10.3390/antiox11112226

**Published:** 2022-11-11

**Authors:** Gry Freja Skovsted, Josephine Skat-Rørdam, Amalie Pihl Frøkiær, Henrik Elvang Jensen, Pernille Tveden-Nyborg, Jens Lykkesfeldt

**Affiliations:** 1 Section of Experimental Animal Models, Department of Veterinary and Animal Sciences, University of Copenhagen, Ridebanevej 9, 1870 Frederiksberg, Denmark; 2 Section of Pathobiological Sciences, Department of Veterinary and Animal Sciences, University of Copenhagen, Ridebanevej 3, 1870 Frederiksberg, Denmark

**Keywords:** vitamin C, atherosclerosis, coronary arteries, guinea pig model, wire myography

## Abstract

Vitamin C (vitC) deficiency has been associated with an increased risk of cardiovascular disease; while several putative mechanistic links have been proposed, functional evidence supporting a causal relationship is scarce. In this study, we investigated how vitC deficiency affects coronary artery vasomotor function and the development of coronary atherosclerotic plaques in guinea pigs subjected to chronic dyslipidemia by a high-fat diet regime. Female Hartley guinea pigs were fed either a control (low-fat diet and sufficient vitC) (N = 8) or a high-fat diet with either sufficient (N = 8) or deficient (N = 10) vitC for 32 weeks. Guinea pigs subjected to the high-fat diet developed significant atherosclerotic plaques in their coronary arteries, with no quantitative effect of vitC deficiency. In isolated coronary arteries, vasomotor responses to potassium, carbachol, nitric oxide, or bradykinin were studied in a wire myograph. Carbachol, bradykinin, and nitric oxide mediated relaxation in the coronary arteries of the control group. While vasorelaxation to carbachol and nitric oxide was preserved in the two high-fat diet groups, bradykinin-induced vasorelaxation was abolished. Interestingly, bradykinin induced a significant contraction in coronary arteries from vitC-deficient guinea pigs (*p* < 0.05). The bradykinin-induced contraction was unaffected by L-NAME but significantly inhibited by both indomethacin and vitC, suggesting that, during vitC deficiency, increased release of arachidonic acid metabolites and vascular oxidative stress are involved in the constrictor effects mediated by bradykinin. In conclusion, the present study shows supporting evidence that poor vitC status negatively affects coronary artery function.

## 1. Introduction

Ischemic heart disease is the most common cause of death worldwide [1]. Although it is a multifactorial condition, the progression of atherosclerosis is known to involve inflammation and oxidative damage, which are often combined with a long-term diet high in fat, cholesterol, and sugars as well as a sedentary lifestyle [2]. Representing a cardiovascular consequence of a chronic state of malnutrition, effects of dietary supplementation on disease and recovery have received increased attention (recently reviewed in [3]). Vitamin C (vitC) is a powerful antioxidant and has been shown to effectively prevent lipid oxidation in plasma [4]. However, in addition to its unspecific role as a low-molecular-weight antioxidant, vitC has also been shown to be a cofactor for several metabolic pathways necessary for vascular health [5]. This includes recycling of tetrahydrobiopterin necessary for nitric oxide production, HIF hydroxylation which is important in angiogenesis and repair response, TET regulation involved in the phenotypic switch of vascular smooth muscle cells, and cholesterol excretion to lower LDL, to mention just a few [6,7].

Several large epidemiological studies and meta-analyses have shown that poor plasma vitC is an independent risk factor for the development of ischemic heart disease and stroke [8,9,10]. While some supporting evidence from intervention studies—e.g., showing the lowering of blood pressure by vitC supplementation—is available [11], the major studies investigating the effect of vitC supplementation on the risk of cardiovascular morbidity and mortality have generally not supported the link between vitC administration and lower disease risk [12]. The possible reasons for this apparent discrepancy between cohort studies and controlled trials have been reviewed in detail elsewhere [13]. However, collectively, the evidence suggests that the putative long-term effect of vitC on cardiovascular health is particularly related to a state of deficiency [14]. In addition, obesity is directly associated with an increased risk of cardiovascular disease [15,16,17], and is linked to an intake high in fat and sugars, which is in turn associated with low vitC status [18,19].

Experimental evidence supporting the role of vitC in vasomotor function is considerable and has shown that vitC infusion increases vasodilation and blood flow, and lowers vasoconstriction following, e.g., methacholine or acetylcholine infusion in humans [20]. Additionally, increased brachial artery dilation has been observed in coronary artery disease patients after oral supplementation compared with placebo [21,22]. More recently, oral vitC supplementation has been shown to restore endothelial function during acute inflammation in young and older adults [23]. However, the mechanisms underlying these effects remain largely undisclosed. Moreover, as most mammals are protected from developing vitC deficiency by their endogenous vitC synthesis, most preclinical models are inept in investigating the putative effects of vitC status in vivo.

Importantly, guinea pigs provide one of the only natural models of diet-induced vitC deficiency, displaying both construct and face validity towards humans. Moreover, guinea pigs display a high degree of similarity with human hepatic lipid metabolism and transport when subjected to a high-fat/high-cholesterol diet [24,25,26,27,28]. Together, these features highlight the guinea pig model as a unique animal model—with a high translational potential—for studying the in vivo effects of diet-induced vitC deficiency and dyslipidemia. Moreover, when comparing species differences in the vasodilator effect of bradykinin on coronary arteries, isolated guinea pig hearts display a significantly higher sensitivity compared with, e.g., rats, rabbits, cats, and dogs, suggesting that the guinea pig provides a putatively superior model for studying effects in coronary vasculature [29]. In young and otherwise healthy guinea pigs placed on a standard control diet, vitC deficiency was shown to alter coronary vessel diameter and decrease contractility when measured by wire myography [30].

The wire myograph is a validated method for evaluating vascular function of coronary vessels ex vivo [31,32]. The technique includes mounting freshly harvested vessel segments on steel wires in a myograph organ bath (with physiologically relevant pH and oxygen levels), whilst introducing pharmacological components to contract the vascular smooth muscle cells in a dose-dependent manner. By recording the induced (or reduction in) tension—as a measure of contractive force—vascular competence can be assessed and related to specific pharmacological targets, thereby identifying the putative mechanisms involved in the elicited response. In addition, the technique permits one to distinguish between different contributions to vascular reactivity by comparing induced responses by several compounds on precontracted arterial segments [32]. In the present study, precontracted vessels segments were exposed to different active agents—namely carbachol (cholinergic agonist), bradykinin (endothelial vasodilator), sodium nitroprusside (nitric oxide donor), L-NAME (inhibitor of endothelial nitric oxide synthase (eNOS)), and indomethacin (cyclic oxide (COX) inhibitor)—to determine the potential pathways involved in changes of vascular contractility and subsequent function.

Consequently, this study explored the effect of vitC deficiency combined with a high-fat/high-cholesterol diet (also recognized as a “westernized” or atherogenic diet regime) on vascular competence; this was evaluated using functional outcome measures of coronary artery vasomotor responses and the development of coronary atherosclerotic plaques in a guinea pig model.

## 2. Materials and Methods

### 2.1. Materials

Carbamylcholine chloride (C4382-1G), indomethacin (I7378-5G), Nω-nitro-l-arginine methyl ester hydrochloride (N5751), L-ascorbic acid (95209), 2-phosphascorbic acid (number), and bradykinin (B3259-5MG) were supplied by Sigma-Aldrich (St. Louis, MO, USA).

### 2.2. In Vivo Model—Design and Diet

Animal experiments were approved by the Danish Experimental Animal Inspectorate (License No: 2018-15-0201-01591) and were in accordance with European legislation on animal experimentation (Directive 2010/63/EU on the protection of animals used for scientific purposes).

**Figure 1:**
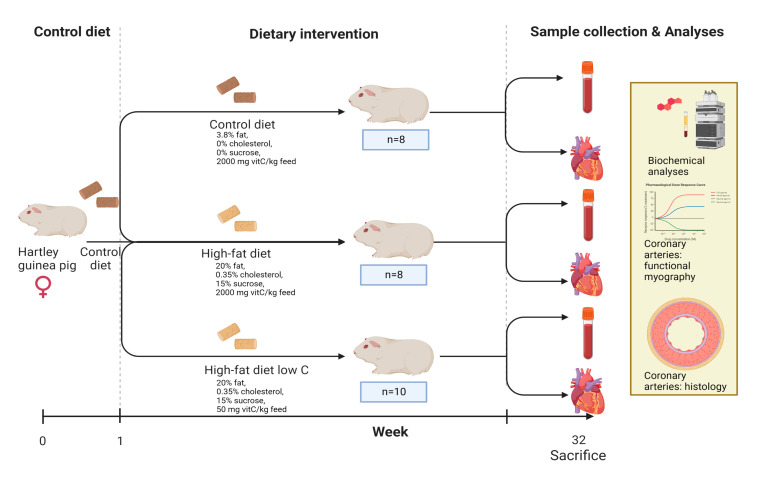
Experimental design of in vivo study. Created using Biorender.

The present investigation was performed on a subset of animals as part of a larger controlled study on nonalcoholic steatohepatitis, the results of which have been published elsewhere [33]. Thus, the full experimental protocol of the in vivo study was described in detail previously [33]. In short, 26 female guinea pigs weighing between 301 and 350 g (Charles River Laboratory, Lyon, France) were subjected to one week of acclimatization period on the control diet; then, they were randomly allocated into three weight-stratified groups: CTRL (N = 8), HFD (N = 8), and HFDLoC (N = 10; Figure 1). The diets consisted of 3.8% fat, 0% cholesterol, 0% sucrose, and 2000 mg vitC/kg feed (CTRL); 20% fat, 15% sucrose, 0.35% cholesterol, and 2000 mg vitC/kg feed (HFD); and 20% fat, 15% sucrose, 0.35% cholesterol, and 50 mg vitC/kg feed (HFDLoC). The animals remained on the diets for 32 weeks. The HFD and HFDLoC groups received feed ad libitum, whereas the CTRL group was pair-fed to the HFD group to avoid excessive caloric compensation in the CTRL group. All groups had ad libitum access to water and access to fixed amounts of hay throughout the study period. All diets were chow-based with vitC provided in the form of phosphorylated ascorbate (Stay-C); the content was confirmed by post-production analysis from the supplier (Ssniff Spezialdiäten, Soest, Germany). For the HFDLoC group, a vitC level of 50 mg/kg feed was obtained by titration, mixing feed containing 100 mg/kg feed with 0 mg vitC/kg. This has previously been shown to induce vitC deficiency without symptoms of scurvy in guinea pigs [30,34]. Body weights were measured once weekly, and animal welfare was monitored daily by caretakers. No changes in behavior or clinical indications of disease or severe vitC deficiency (scurvy) were recorded. After 32 weeks on the diets, all animals were euthanized. Prior to euthanasia, animals were semi-fasted overnight. Pre-anesthesia was induced with 1.25 mL/kg Zoletil-mix (125 mg Tiletamin, 125 mg Zolazapam (Zoletil 50 Virbac Laboratories, Carros, France) + 200 mg xylazin (Narcoxyl vet 20 mg/mL; Intervet International, Boxmeer, Holland) + 7.5 mg butorphanol (Torbugesic vet 10 mg/mL; Scanvet, Fredensborg, Denmark)). The anesthetized animal was then placed on isoflurane (3–5%) and, upon disappearance of inter-digital reflexes, intracardial blood was collected for vitC, free fatty acids (FFA), triglycerides (TG), and total cholesterol (TG), as previously described [35,36,37].

### 2.3. Coronary Artery Function—Endothelial and Smooth Muscle Function

The heart was transferred to ice-cold physiological buffer (PSS) (composition in mM: 119 NaCl, 4.7 KCl, 2.5 CaCl2, 1.2 MgSO4, 1.2 KH2PO4, 25 NaHCO3, and 5.5 glucose; pH 7.4) gassed with CO_2_ bioair 5% (containing 5% CO_2_/21%O_2_ in nitrogen, HiQ^®^ Specialty Gases Mixture Finder, Linde AD, Pullach, Germany). From each heart submerged in buffer, two–three segments of the left anterior descending (LAD) coronary artery were dissected from the surrounding myocardial and connective tissues. The segments were cut into 2 mm-long circular segments and mounted between two 40 µm stainless steel wires in a wire myograph organ bath (610 M, Danish Myo Technology A/S, Aarhus, Denmark) containing cold PSS. Isometric force was recorded, displayed, and analyzed by a PowerLab/35 system and LabChart Pro 8 (version 8.1.16) (AD Instruments, Oxford, UK). Artery segments were equilibrated for 30 min while the buffer was slowly heated to 37 °C. Then, each segment was normalized to a circumference corresponding to 90% of its circumference at a pressure of 13.3 kPa. After normalization and another 15 min equilibration period in PSS, the segments were challenged 2–3 times with 125 mM potassium (similar composition as the above PSS buffer, except that NaCl was exchanged with KCl on equimolar basis) to measure the vasoconstrictor capacities of the arteries. Only segments with a potassium-induced contraction >0.5 N/m were included in the study. As some segments failed to reach this criterion, group sizes (N-number) and n-values (segment number) may differ.

A stable pre-constriction was obtained with 40 mM potassium (40 mM KCl containing PSS) before cumulative addition of carbachol (1 nM–0.5 mM), bradykinin (10 pM–0.5 µM) or sodium nitroprusside (1 nM–0.1 mM). In order to further elucidate the bradykinin vasomotor responses, bradykinin concentration–response curves were acquired either in the absence (controls) or in the presence of the COX-inhibitor indomethacin (10 µM), the eNOS inhibitor L-NAME (10 µM), or vitC (a mixture containing 75 µM L-ascorbic acid and 100 µM 2-phosphoascorbic acid). Sodium nitroprusside was used to test endothelium-independent vasorelaxation. Immediately after dissecting LAD segments for functional studies, the remaining hearts were immersion fixated in 10% neutral buffered formalin for 3–5 days.

### 2.4. Histology

Formalin-fixated hearts were divided transversely at the basis of the left atrium, and transversely sectioned specimens of the coronary arteries were embedded in paraffin and sectioned at 4 μm. Sections were stained with hematoxylin and eosin (H&E), periodic acid Schiff (PAS), Toluidine blue, or Congo Red. After staining, sections were scanned into a computer with Axio Scan.Z1 (ZEISS, Birkerød, Denmark) and were histologically evaluated.

### 2.5. Quantification of Vascular Remodeling

Quantification of vascular remodeling was performed using Visiopharm-integrated software VIS (Visiopharm, Hoersholm, Denmark) on PAS and H&E-stained heart sections. All histopathological scorings were performed in a randomized and blinded manner. From each heart, twelve (non-occluded and partly occluded) arteries were selected by diameter size (as defined by the external lamina) commencing with the largest and continuing systematically downward in diameter size throughout the histological sections; all completely occluded arteries were selected for analysis. External and internal elastic laminas and the inner layer of the intima were outlined, and the area of each layer (lumen, intima, and media) was determined. From the area of each layer, the diameter was calculated using the following formula: diameter_total_ = 2 × (√(area/3.14)). The diameter of individual layers was calculated as follows: media (diameter_media_= diameter_total-media_-diameter_total-intima_); intima (diameter_intima_= diameter_total-intima_-diameter_lumen_); lumen (diameter_lumen_). The relative thickness of the media and the intima and the diameter of the lumen relative to the total diameter (diameter_total-media_) were calculated in percent for each vessel.

### 2.6. Statistics

VitC status and dyslipidemia markers were analyzed as described in Table 1. Following tests for homogeneity of variances, myograph studies were analyzed by two-way ANOVA and Tukey’s multiple-comparisons test, mixed-effects analysis, and Šídák’s comparisons test using GraphPad Prism 9.3.1. Histological data were evaluated by one-way ANOVA and Tukey’s multiple-comparisons test by using RStudio 2022.02.01. Pearson’s correlation analysis was used to assess the relationship between histological and biochemical endpoints. A *p*-value of less than 0.05 was considered statistically significant.

## 3. Results

### 3.1. VitC Status and Biomarkers of Vascular Dyslipidemia

Plasma concentrations of vitC and markers of dyslipidemia are shown in Table 1. The vitC deficiency of the HFDLoC group compared with the HFD group was confirmed (*p* < 0.001), as were the expected effects of the high-fat diet discussed in detail elsewhere [33].

### 3.2. General Vasomotor Responses

Potassium (125 mM) evoked contraction and was used as internal control for each artery segment. Potassium-induced contractions and internal diameters did not differ significantly between the three diet groups (Table 2).

### 3.3. Carbachol-Mediated Vasoactive Responses

**Figure 2:**
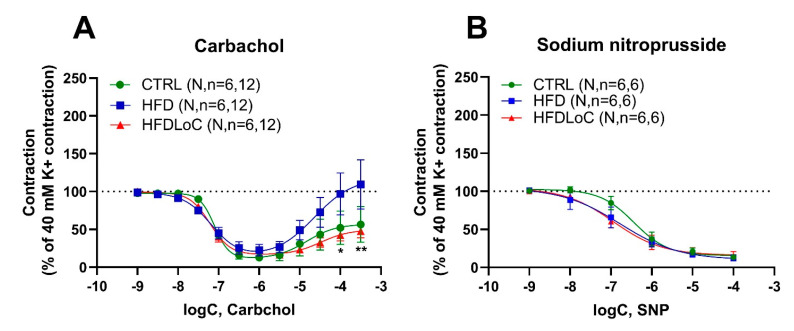
Wire myography studies in coronary arteries precontracted with 40 mM potassium and stimulated with (**A**) the muscarinic receptor agonist (carbachol) or (**B**) the nitric oxide donor (sodium nitroprusside) to induce endothelium-independent relaxation. (**A**) Carbachol induced similar relaxation in coronary arteries of the control (CTRL), HFD, and HFDLoC groups, whereas arteries from the HFD group contracted significantly higher concentrations of carbachol compared with the HFDLoC group. (**B**) Sodium-nitroprusside-induced relaxation was not different between the coronary arteries of the three diet groups. Mean ± SEM, * *p* < 0.05, ** *p* < 0.01 HFDLoC vs. HFD determined by 2-way ANOVA and Tukey’s multiple-comparisons test. N, number of animals; *n*, number of investigated segments.

In coronary arteries, which were stably precontracted by potassium, the muscarinic receptor agonist carbachol induced a biphasic response, producing relaxation at lower concentrations, followed by moderate contraction at higher concentrations > 1 µM (Figure 2A). While carbachol-mediated relaxation was not significantly different between the three diet groups, carbachol-mediated contractions were notably attenuated by vitC deficiency compared with the HFD group, and non-significantly different from the CTRL group.

### 3.4. Endothelium-Independent Relaxation by Sodium Nitroprusside

Sodium nitroprusside—a NO donor promoting NO-mediated vasodilation—induced concentration-dependent relaxation in a similar pattern in precontracted coronary arteries (Figure 2B) with no significant differences between the diets.

### 3.5. Bradykinin-Mediated Vasoactive Responses

**Figure 3:**
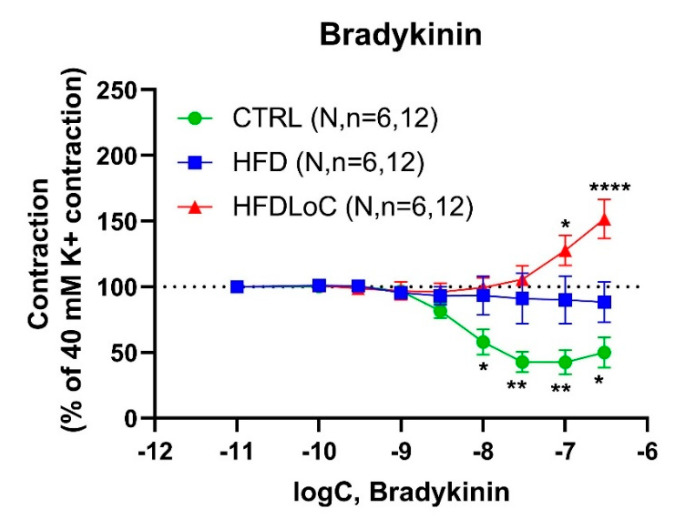
Concentration–response curves to bradykinin in guinea pig coronary arteries contracted by 40 mM K^+^. Results are expressed as percent change from the plateau contraction to 40 mM K^+^. * Significantly different from HFD. Mean ± SEM, * *p* < 0.05, ** *p* < 0.01, **** *p* < 0.0001 HFDLoC or CTRL vs. HFD determined by 2-way ANOVA and Tukey’s multiple-comparisons test. N, number of animals; *n*, number of investigated segments.

Bradykinin (associated with endothelial vasodilation) induced a concentration-dependent relaxation with an Emax = 56.0 ± 9.9 (% relaxation) and pEC_50_ =8.3 ± 0.1 in precontracted coronary arteries from guinea pigs from the CTRL group. Interestingly, HFD abolished bradykinin-mediated relaxation; with vitC deficiency, bradykinin induced substantial contractions of Emax = 74.0 ± 21.6 (% contraction) and pEC_50_ =7.3 ± 0.3 (Figure 3).

### 3.6. Effects of L-NAME, Indomethacin, and VitC on Bradykinin-Mediated Vasoresponses

To evaluate the contribution of NO, prostanoids, and oxidative stress to bradykinin-induced vasodilation and vasoconstriction, bradykinin concentration–response curves were recorded in the presence of either the COX inhibitor, indomethacin (10 µM), the NOS inhibitor, L-NAME (10 µM) or the free radical scavenger, vitC (75 µM L-ascorbic acid and 100 µM 2-phosphoascorbic acid), respectively. The inhibitors/scavengers were added 15 min prior to stimulation with bradykinin.

**Figure 4:**
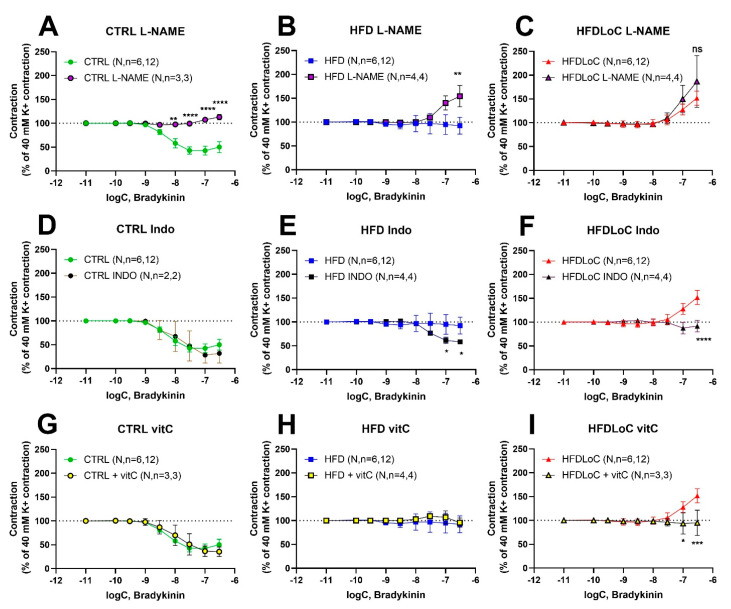
Concentration–response curves to bradykinin obtained in the absence or (**A**–**C**) presence of L-NAME (10 µM), (**D**–**F**) indomethacin (10 µM), and (**G**–**I**) vitC (75 µM L-ascorbic acid and 100 µM 2-phosphoascorbic acid). Results are expressed as percent change from the plateau contraction to 40 mM K^+^. Mean ± SEM, * *p* < 0.05, ** *p* < 0.01, *** *p* < 0.001, **** *p* < 0.0001 without vs. with L-NAME, Indo, or vitC, determined by mixed-effects analysis and Šídák’s comparisons test. N, number of animals; *n*, number of investigated segments.

In segments from the CTRL animals, the NO-inhibitor L-NAME abolished bradykinin-induced relaxation, while COX inhibition by indomethacin and vitC (targeting free radical scavenging) had no effect on bradykinin responses (Figure 4A,D,G). In contrast, NO inhibition with L-NAME provoked a significant bradykinin-induced contraction in segments from HFD guinea pigs, while COX inhibition by indomethacin elicited a bradykinin-induced relaxation (Figure 4B,E). Free radical scavenging by vitC supplementation had no effect (Figure 4H). In segments from VitC-deficient guinea pigs, the presence of L-NAME had no effect on bradykinin-induced contraction, whereas indomethacin-mediated COX inhibition and vitC supplementation (free radical scavenging) both attenuated bradykinin-induced contractions significantly (Figure 4C,F,I).

### 3.7. Histology

**Figure 5:**
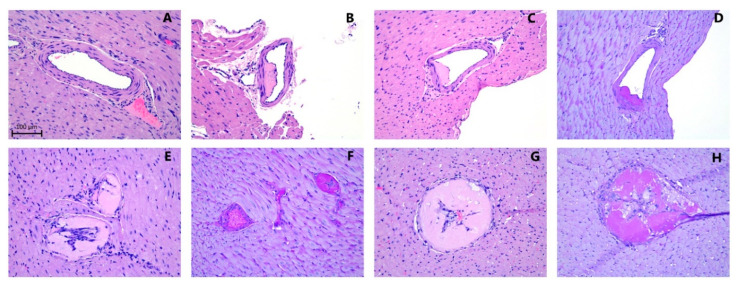
Representative examples of coronary arteries from guinea pigs fed with (**A**) control diet sufficient with vitC (CTRL); atherosclerotic lesions from coronary artery sections (**B**,**E**,**F**) following a fed high-fat diet sufficient in vitC (HFD) or (**C**,**D**,**G**,**H**) from animals fed a high-fat diet with low vitC (HFDLoC); (**A**–**C**,**E**,**G**) H&E and (**D**,**F**,**H**) PAS staining. Scale bar =100 µm.

**Figure 6:**
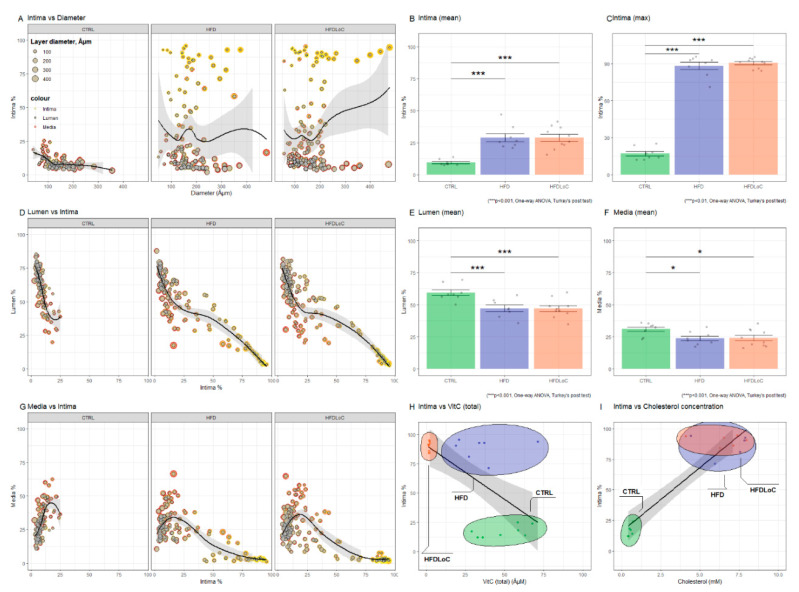
Quantification of intima, lumen, and media as percent of vessel diameter calculated from lamina elastic externa. (**A**) Intima % versus diameter shows increased intima (above approximately 25%) in coronary arteries from HFD and HFDLoC animals. (**B**) Average of all measured artery segments from each animal and (**C**) average of the most stenotic arteries from each animal shows that the average relative intima thickness and the average of the most stenotic arteries from each animal is significantly increased in HFD and HFDLoC compared with CTRL animals. (**D**) Lumen % versus Intima % shows a negative linear relationship between relative intima thickness and lumen. Increasing intima > 25% further decreases the lumen in HFD and HFDLoC. (**G**) Media % versus intima % shows a positive correlation between intima and media in CTRL, in contrast to HFD and HFDLoC animals, where increasing intima >25% results in decreasing media, suggesting an atrophic remodeling. The average of (**E**) the arteries with smallest lumen and (**F**) the smallest media from each animal shows no significant difference between the groups. (**H**) Intima (top) % versus vitC (total) concentration shows a significant negative correlation between intima% and vitC concentration (Pearson’s r = −0.36, *p* < 0.05); (**I**) Intima (top) % versus cholesterol concentration shows a significant positive correlation (Pearson’s r = 0.54, *p* < 0.01). Columns represent average ± SEM of each animal. * *p* < 0.05, *** *p* < 0.001 HFD and HFDLoC vs. CTRL determined by one-way ANOVA and Tukey’s post hoc test.

Atherosclerotic plaques within the coronary arteries are presented under histomorphological tests as either homogenous deposits of PAS-positive material or PAS-positive material together with vacuole-containing cells. The plaques were negative in Congo Red and Toluidine blue staining. Representative images of vascular histomorphology are shown in Figure 5. Quantification of the vascular characteristics is shown in Figure 6.

## 4. Discussion

In the present study, we showed that guinea pigs fed with a high-fat diet develop profound PAS-positive atherosclerotic plaques totally or partly occluding the lumen regardless of vitC status. Furthermore, vitC deficiency impaired coronary artery function as measured by bradykinin-induced relaxation in precontracted coronary arteries. Finally, vitC deficiency exacerbated coronary artery dysfunction with enhanced contractions in response to bradykinin. Because the addition of vitC or indomethacin reversed the constrictor responses in a similar pattern, our data support the idea that enhanced oxidative stress and release of vasoconstrictor prostaglandins may be the underlying causes of the bradykinin-induced contractions in vitC-deficient guinea pigs.

Reviewing the current findings, the response to a chronic state of diet-induced dyslipidemia in guinea pigs manifested in the coronary vasculature as significant atherosclerotic depositions. Accompanying alterations in dilatory competence ex vivo functional tests clearly supported the induction of significant effects in the vasomotor responses of coronary arteries, even though all animals remained clinically healthy on inspection. Several studies in experimental animal models and in humans have established an association between a high-fat/high-cholesterol diet and the promotion of atherosclerosis [38,39]; however, experimental animal models often require additional insults, such as genetic manipulation (e.g., *ApoE*^-/-^ mice) or excessive dietary regimes, to promote pathological changes in the vascular wall [40,41]. In this regard, the current findings strongly support the relevancy of the guinea pig model in exploration of the direct effects of dietary regime on LAD. While a low vitC status displayed a negative correlation with the relative intima thickness, most measures did not reveal significant differences between the high- and low-vitC HFD groups, rendering the potential isolated effect of vitC deficiency on the histopathological changes largely undetectable. However, this is not surprising, because the primary driver of the observed vascular damage is most likely the circulating lipids and—more so—cholesterol, in the form of ox-LDL, which subsequently lodges in the vascular wall and provides a basis for developing atheromas [42,43]. A distinctive role of vitC deficiency is therefore highly plausible, although detectable effects may well have been overpowered by the high-fat diet.

In guinea pigs, plasma ascorbate directly predicts the ratio of BH4:BH2 (tetra- and di-hydrobiopterin, respectively) with vitC deficiency leading to increased levels of BH2 and consequently altering BH4:BH2 homeostasis [44]. In vivo, BH4 functions as a co-factor of endothelial nitric oxide synthetase (eNOS), supporting eNOS coupling and thereby promoting NO formation instead of superoxide formation [45,46]. In turn, decreased levels of NO are associated with a compromised endothelial function [47]. This links vitC deficiency to biopterin imbalance and eNOS uncoupling, with putative negative consequences on the vascular endothelium and promoting endothelial dysfunction [26]. A state of endothelial dysfunction is prompted by changes in vasoactive signaling altering vasoconstrictive and vasodilating mechanisms (often linked to NO availability) [48,49]. Interestingly, patients with coronary artery disease displayed impaired bradykinin-induced vasodilation specifically at atherosclerotic sites; meanwhile, this response was preserved at normal sites [50]. Bradykinin is a vasodilator nonapeptide that is formed by cleavage of high-molecular-weight (HMW) kininogens through the tissue and plasma kallikrein enzymes. Although HMW is mainly synthesized in hepatocytes and released into plasma, there is also a local production of bradykinin in the heart [51]. Endogenous bradykinin regulates coronary blood flow [52] and can be particularly important during atherosclerotic conditions and during cardiac ischemia, where production of bradykinin has been shown to be increased [51]. Basal bradykinin release in the heart has been shown to induce an eightfold increase in ischemia, suggesting that endogenous bradykinin can potentially induce coronary contraction at atherosclerotic sites [51]. Additionally, bradykinin induces vasoconstriction at atherosclerotic sites [50]; accordingly, it potentially worsens the ischemic condition in the myocardium downstream of the stenotic plaque.

In the present study, precontracted coronary arteries from guinea pigs fed a high-fat diet (both vitC-sufficient and -deficient diets) relaxed to carbachol in a similar way to control animals. Further increased carbachol concentrations (>1 µM) induced a contraction in arteries from HFD (vitC-sufficient diet). Interestingly, this secondary contraction was significantly attenuated in the arteries of vitC-deficient guinea pigs. We have previously shown that carbachol elicits its effect via muscarinic receptors in guinea pigs, since atropine completely blocks the vasomotor response [30]. Furthermore, vitC deficiency attenuated carbachol-induced contraction in guinea pig coronary arteries; this effect may be due to altered muscarinic receptor activity and/or altered arachidonic acid metabolite release [30]. It may therefore be speculated that an apparent absence of high-dose carbachol contractility in HFDLoC could be due to a reduced availability of muscarinergic receptors in the vascular wall (M3 receptors), either because of a reduced number of receptors or a decrease in sensitivity. This would then support a vitC-deficiency-evoked state of vascular dysfunction in HFDLoC animals, even though the measured contractive force was not significantly different from that of the healthy controls.

In isolated canine coronary arteries, bradykinin-induced relaxation was abolished by pretreatment with L-NAME [9], inhibited by depolarization with potassium, and inhibited by removal of the endothelium [10]; meanwhile, pretreatment with indomethacin did not alter the vasorelaxant response [10,11]. This suggests that bradykinin-induced relaxation is mediated by eNOS and endothelium-derived hyperpolarizing factor and not by arachidonic acid metabolites, at least not in this species.

In agreement with a functional link between bradykinin and eNOS, the present study disclosed that bradykinin induced an eNOS-mediated relaxation in the precontracted coronary arteries of control guinea pigs on a low-fat vitC-sufficient diet. This is in accordance with previous studies showing that bradykinin is a potent vasodilator and increases coronary flow in isolated guinea pig hearts in a concentration-dependent manner [29,53]. In isolated guinea pig hearts, 1 nM bradykinin doubled and even tripled the coronary flow, which reached 8–9 mL/min compared with the basal flow, and reached approximately half of the maximal vasodilatory capacity at approximately 20 mL/min [29,53].

Kinins, kallidin, bradykinin, and their metabolites (des-arg9-bradykinin and des-arg10-kallidin) mediate vascular responses through stimulation of two distinct receptor subtypes, B2 and B1. While B2 receptors are constitutively expressed, particularly in endothelial cells, and have preferred affinity for the native kinins (bradykinin and kallidin), the B1 receptors are upregulated or de novo expressed following tissue injury and inflammation and have a preferred affinity for metabolites (des-arg9-bradykinin and des-arg10-kallidin) [54,55]. In humans, the B2 receptor antagonist Hoe 140 decreased coronary blood flow and increased coronary vascular resistance; these findings imply that endogenous bradykinin may be central to the regulation of coronary tone [52]. In a canine model, the vasodilator effects of isolated coronary arteries and open-chest coronary blood measurements were unaffected by B_1_ receptor antagonists, but were attenuated by competitive B_2_ receptor antagonists [54,55]. Collectively, this suggests that bradykinin as a key factor in the regulation of coronary blood flow through its binding to B2 (and B1) receptors in the coronary vascular endothelium. Moreover, bradykinin displays a protective role in acute ischemic cardiac injury and subsequent myocardial response to reperfusion, proposing bradykinin-mediated signaling as pivotal in the regulation of blood flow to the heart muscle and in the protection against endothelial dysfunction associated with the progression of cardiovascular disease [56,57].

Interestingly, in atherosclerotic coronary arteries—while the relaxation to bradykinin was abolished—we found that acetylcholine-induced relaxation was preserved. In human coronary epicardial and resistance arteries, bradykinin induces dilatation and increases coronary blood flow [58]. Intracoronary infusion of bradykinin in doses 0.6 and 2.0 µg/min raised coronary blood flow from 41 ± 27 to 79 ± 46 and 107 ± 65 mL/min, respectively. Bradykinin-induced dilatation was inhibited by the NO synthesis inhibitor L-NMMA, and was increased by the ACE inhibitor enalaprilat, supporting the idea that bradykinin induces dilation via NO release and that bradykinin is metabolized by ACE in humans [58]. A study by Feher et al. also found that bradykinin-induced dilation was diminished in the coronary arteriolar segments from high-fat fed rats, compared with controls. This diminished dilatation was restored after in vitro administration of the ACE inhibitor captopril. This suggests that HFD and obesity leads to increased tissue ACE activity in the coronary arteries, which increases the proteolytic degradation of bradykinin and thus attenuates its dilatory duration of action [59].

## 5. Conclusions

In conclusion, the current study shows severe changes to LAD histomorphology in HFD guinea pigs. Subsequent ex vivo analyses in high-fat-diet-fed animals demonstrate changes in vasomotor competence depending on dietary status (low-fat diet controls versus high-fat diet group) with a clear effect of vitC deficiency on bradykinin-induced vasodilation. This suggests a putative and yet undisclosed direct role of vitC in the bradykinin-mediated regulation of coronary vasomotor function during chronic dyslipidemia and subsequent atherosclerosis.

## Figures and Tables

**Table 1:** Plasma vitamin C concentrations and markers of dyslipidemia in the guinea pigs.

Plasma Marker	CTRL	HFD	HFDLoC
Number of animals	8	10	10
VitC (µmol/L)	48.17 (34.91–58.63)	33.92 (21.89–39.55) *	1.76 (1.40–1.92) ^###^
FFA (mmol/L)	0.38 ± 0.22	0.54 ± 0.14	0.5 ± 0.14
TG (mmol/L)	0.65 (0.54–0.82)	0.54 (0.45–0.66)	0.64 (0.53–0.69)
TC (mmol/L)	0.53 (0.43–0.66)	7.42 (5.94–7.84) ***	6.88 (6.18–8.97)

HFD animals was compared to the CTRL group to test the effect of high-fat diet, while the HFDLoC group was compared to the HFD to identify effects of vitC deficiency. Data are presented as means ± SD (parametric data) or medians with Q25-Q75 values (log-transformed data) and analyzed by t-test except for vitC that was analyzed by using a t-test with Welch correction due to unequal variances. Difference from LFH: * *p* < 0.05, *** *p* < 0.001. Difference from HFD: ^###^ *p* < 0.001. VitC, vitamin C; FFA, free fatty acids; TG, triglycerides; TC, total cholesterol. Data adapted from ref [33].

**Table 2:** Maximal potassium-induced contraction and calculated diameter of artery segments included in myograph studies. Data are presented as means ± SEM.

	CTRL (N, *n* = 6, 13)	HFD (N, *n* = 6, 12)	HFDLoC (N, *n* = 6, 12)
Potassium (125 mM) (mN)	9.05 ± 1.33	12.17 ± 1.29	10.09 ± 1.26
Diameter (µm)	363 ±3 0	437 ± 30	364 ± 37

mN, milli newton; N, number of animals; *n*, number of investigated segments.

## Data Availability

Data and material supporting the conclusions of this article can be requested from the authors.
